# Silica Nanoparticles Block Natural Genetic Transformation in *Acinetobacter baylyi* ADP1

**DOI:** 10.1021/acsomega.5c06566

**Published:** 2025-12-15

**Authors:** Samuel Chetachukwu Adegoke, Ignatius Senyo Yao Yawlui, Dennis LaJeunesse

**Affiliations:** Joint School of Nanoscience and Nanoengineering, Department of Nanoscience, E Gate City Blvd., Greensboro, North Carolina 27402-6170, United States

## Abstract

The prolonged and widespread use of antibiotics has driven the emergence of resistance to many commonly employed drugs, posing a growing global challenge that requires urgent measures to curb its spread. Once resistance develops, horizontal gene transfer facilitates the exchange of genetic materials among various bacterial species, often preceding vertical transmission. Previous work to control horizontal gene transfer and specifically natural transformation within a population of bacteria approached the problem by addressing the bacterial mechanisms required for transformation. In this study, we investigated the possibility of controlling horizontal gene transfer by limiting access to or the availability of environmental DNA to the bacteria. In this study, we investigated the impact of five different sizes of silica nanoparticles (SiO_2_NPs), 20, 80, 120, 200, and 500 nm, and three sizes of gold nanoparticles (AuNPs), 5, 20, and 200 nm, on the natural genetic transformation of *Acinetobacter baylyi* ADP1 (*A. baylyi* ADP1) using both circular and linear environmental DNA (pBTK501) carrying an ampicillin resistance cassette. Our findings reveal that SiO_2_NPs ranging from 120 to 500 nm consistently inhibited transformation events in both M9 and LB media. SiO_2_NPs effectively suppress the natural transformation of *A. baylyi* ADP1 in the presence of circular pBTK501 with a stronger effect on the linear pBTK501. The degree of inhibition was size-dependent, as the 500 nm SiO_2_NPs exhibited the strongest effect. The inhibitory effect of SiO_2_NPs was also found to be dose-dependent: increasing the pBTK501 concentration relative to the SiO_2_NPs diminished the inhibition, while a higher SiO_2_NP-to-pBTK501 ratio resulted in a stronger inhibition. Similarly, the 200 nm AuNPs also displayed a notable inhibitory effect on the natural transformation of *A. baylyi* ADP1. These results, taken together, appear to show the ability of nanoparticles to control natural transformations in *A. baylyi* ADP1. This size-dependent mechanism clearly defines a path to mitigate the spread of resistance evolution both at the hospital and community settings, which hitherto has not been given adequate consideration.

## Graphical abstract

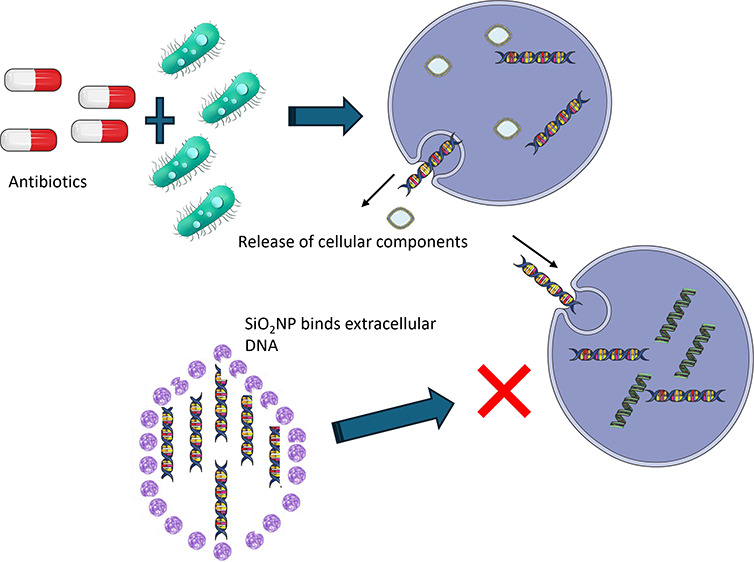

## Introduction

Bacterial resistance to antibiotics is a global health crisis. Antibiotic resistance evolves in microbial populations through the indiscriminate and widespread application of antibiotics in different spheres—clinical medicine, agricultural application, animal production, and within the environment via improper pharmaceutical disposal.^1^ Compounding this evolutionary process is the mechanism of horizontal gene transfer (HGT), which enables microbes to share the evolved antibiotic resistance genes (ARGs).^2–4^ There are three primary mechanisms by which horizontal gene transfer occurs in prokaryotic organisms: conjugation, transformation, and transduction.^2^ While conjugation between two microbes and transduction via viruses involve physical contact between the donors and the recipients, transformation is the constitutive ability of the microbial cell to uptake DNA from its environment.^2,5^ Transformation occurs in all environmental conditions in the presence of high-molecular-weight DNA molecules.^6^ In the case of bacteria, extracellular DNA molecules are captured by cellular appendages called competence pili, which after binding the target DNA subsequently translocate this DNA to the cellular surface. Following this initial interaction, an ATP-dependent process facilitates the degradation of one DNA strand while mediating the transport of the complementary strand into the cytoplasmic compartment.^7^ Upon internalization, the exogenous DNA undergoes genomic integration or plasmid circularization via homologous recombination mechanisms or alternatively may be enzymatically degraded to serve as a nutritional resource for the bacterial cell.^7,8^

Extracellular conditions are critical for facilitating natural transformation; however, little work has been done to demonstrate how control over environmental DNA impacts this process. Recently, researchers have successfully halted bacterial resistance evolution via disrupting the canonical HGT pathways using genetic and synthetic biological approaches. In one study, researchers used synthetically derived peptide-based moieties, competence stimulating protein1-E1A (CSP1-E1A), and competence stimulating protein 2-E1A (CSP2-E1A) to competitively inhibit the ability of *Streptococcus pneumoniae* to acquire the streptomycin resistance gene (*rpsL*) and the capsule gene *cap3A* during a transformation event.^9^ The CSP1-EA1 and CSP2-EA1 peptides function in quorum sensing, triggering cells within a population to become more competent for transformation. Although this work demonstrates that transformation can be controlled by the addition of extracellular agonists, such peptide-based approaches will be difficult to employ under real-world conditions. In this paper, we present work that demonstrates the use of nanoparticles to block the transformation process. *A. baylyi* ADP1 is a naturally and highly competent Gram-negative bacterium, which unlike other naturally competent bacteria does not require a stimulus.^10^ *A. baylyi* ADP1 has demonstrated transformation frequencies as high as 1:1000.^11,12^ SiO_2_NPs have been reported to adsorb DNA molecules including plasmids with strong force;^13–15^ hence, their application in the biomedical arena as drug carriers and gene delivery agents has had established success.^16^ Although SiO_2_NPs have been used as a transfection agent in the fibroblast-like cell line COS-1,^17^ there are limited studies on its use to control natural genetic transformation and the spread of antibiotic resistance genes in a variety of contexts. In this article, we used *A. baylyi* ADP1 as a model to determine the role of the environment in the control of natural genetic transformation. We observed a significant suppression of transformation by *A. baylyi* ADP1 using silica and gold nanoparticles. Furthermore, we demonstrate that nanoparticles suppress transformation by sequestering of environmental DNA and not by perturbation of the bacteria, suggesting that these materials may be used to control transformation and HGT in complex and diverse conditions.

## Results

To determine whether the nanomaterial influences the transformation of environmental DNA by *A. baylyi* ADP1, we assayed the impact of SiO_2_NPs on the transformation efficiency, i.e., the number of transformed (i.e., resistant cells) relative to the total number of cells in the population (Figure 1).^12^ Recent work using artificial sweeteners as carbon sources has demonstrated that changes to the nutritional state of a microbe alter transformation.^19^ Metabolic processes drive the behavior of bacteria and other microbes and often control the state and condition of the cells.^20^ To determine whether nutrient conditions alter the transformation process, we used two different growth media, Luria broth (LB) and M9 minimal medium, supplemented with 2% glucose (GL). GL is a defined synthetic media, and LB is a complex medium containing multiple carbon sources and often with some nutrients in excess.^18^ In either media, we observed no significant changes in *A. bayly*i ADP1 fitness as defined by a growth curve analysis (Figure S1). In both media, *A. baylyi* ADP1 demonstrated a natural transformation.

**Figure 1:**
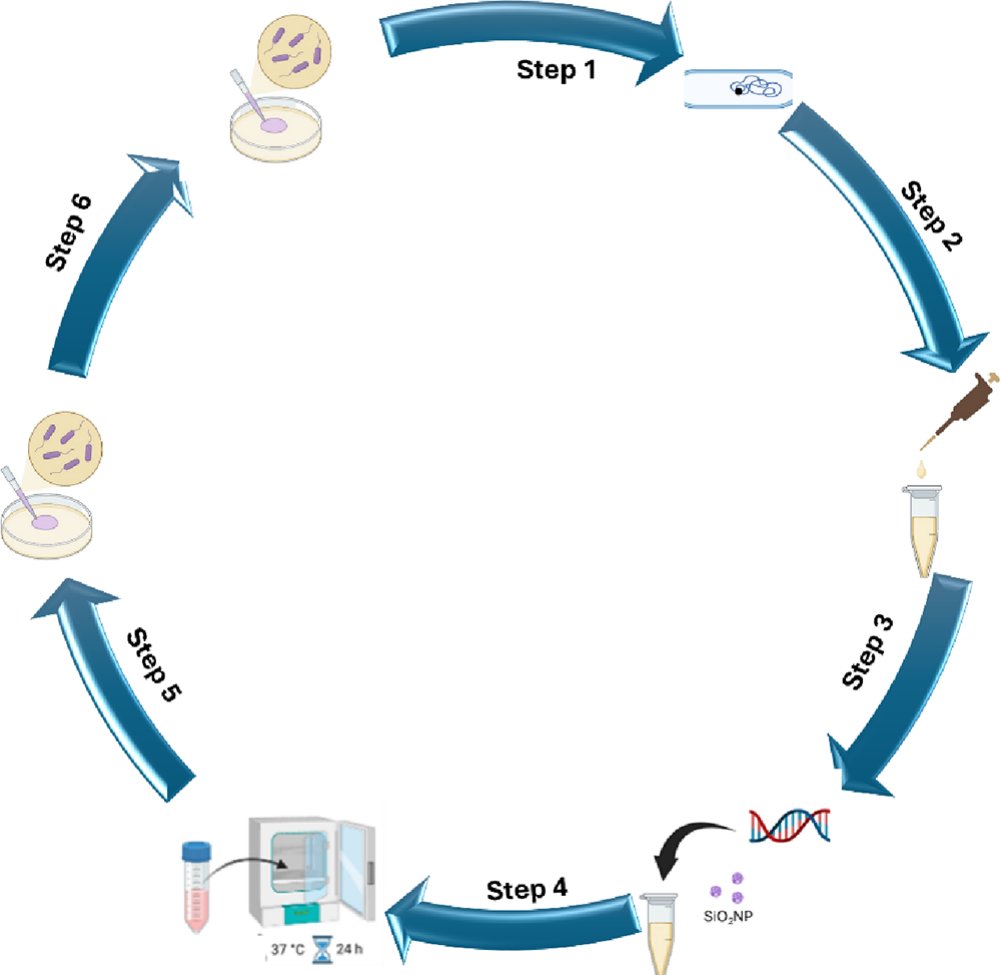
Schematic representation of methodologies and findings. (Step 1) Competent *A. baylyi* ADP1 is grown at 30 °C overnight (OVN). (Step 2) 70 μL of OVN *A. baylyi* ADP1 is added to 930 μL of fresh LB/M9. (Step 3) 250 ng of tDNA (circular and linear) and SiO_2_NP. (Step 4) The reaction mix is grown OVN at 37 °C at 150 rpm. (Step 5) 10^5^ of OVN *A. baylyi* ADP1 are drop-plated on nonselective agar to enumerate the total number of nontransformants and grown for 16 h at 37 °C. (Step 6) 10^5^ of OVN *A. baylyi* ADP1 are drop-plated on selective agar to enumerate the total number of transformants and grown for 16 h at 37 °C. Created with Biorender.com.

To determine the impact of SiO_2_NPs on transformation, we measured the transformation efficiency of *A. baylyi* ADP1 in the presence of SiO_2_NPs from 20 to 500 nm (Figures 2A and 3A). The interaction of silica surfaces with single- and double-stranded DNA has been reported,^21–23^ and we wanted to determine whether these materials interfere with natural transformation. In these experiments, we kept the ratio of the number of the plasmid pBTK501 DNA molecules to SiO_2_NPs at a 1:1 ratio of the number of DNA molecules to nanoparticles (see methods). In the M9 + 2% glucose medium (GL), we observed a systematic reduction in transformation efficiency as the sizes of the nanoparticles increased greater than 80 nm (Figure 2A). In the LB medium, we observed a similar trend (Figure 3A). All SiO_2_NP treatments showed significant reduction in transformation efficiency compared to the positive control (*p* < 0.05); the 500 nm treatment reduced transformation the greatest with a 98-fold reduction in transformation in GL medium (Figure 2B, last column).

**Figure 2:**
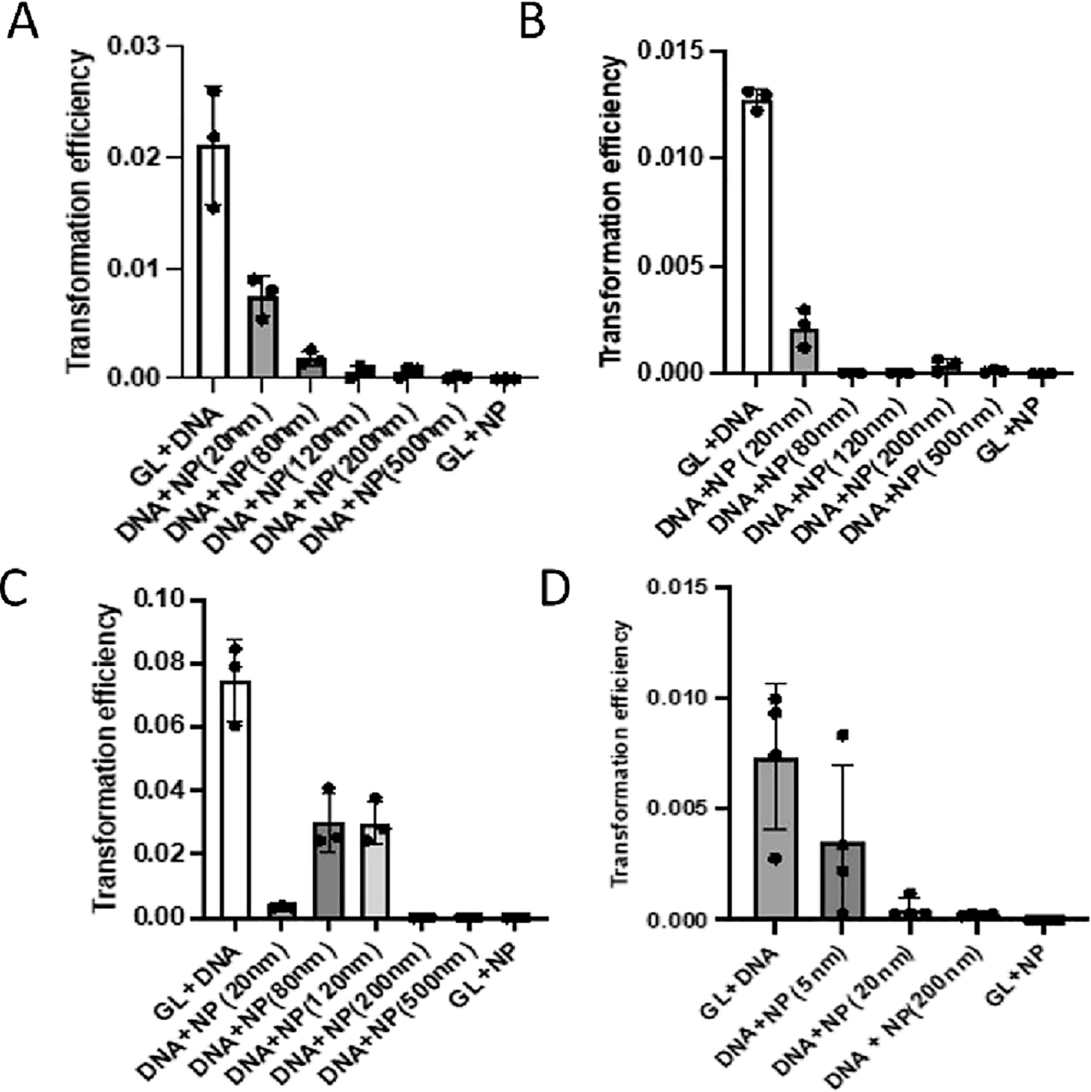
Reduction of the *A. baylyi ADP1* transformation efficiency by silica and gold nanoparticles. All cells were cultured in M9 + 2% glucose minimal medium (GL). Positive controls are in the first column (LB + DNA). (A) Size dependence of SiO_2_NP inhibition of transformation (DNA 1:1 SiO_2_NP); larger particles (columns 5 and 6: 200 and 500 nm SiO_2_NPs) blocked transformation better than smaller particles. (B) Increasing the NP-to-pBTK501 DNA ratio (DNA 1:3 SiO_2_NP) increases blocking of transformation with smaller nanoparticles showing substantial blocking; (C) increasing the pBTK501 DNA-to-NP ratio (DNA 3:1 SiO_2_NP) reduces the blocking of transformation by 80 and 120 nm particles, while the 200 and 500 nm remain effective. Curiously, 20 nm particles become more effective; (D)­AuNPs also reduce *A. baylyi ADP1* transformation efficiency with particles larger than 20 nm blocking more effectively.

**Figure 3:**
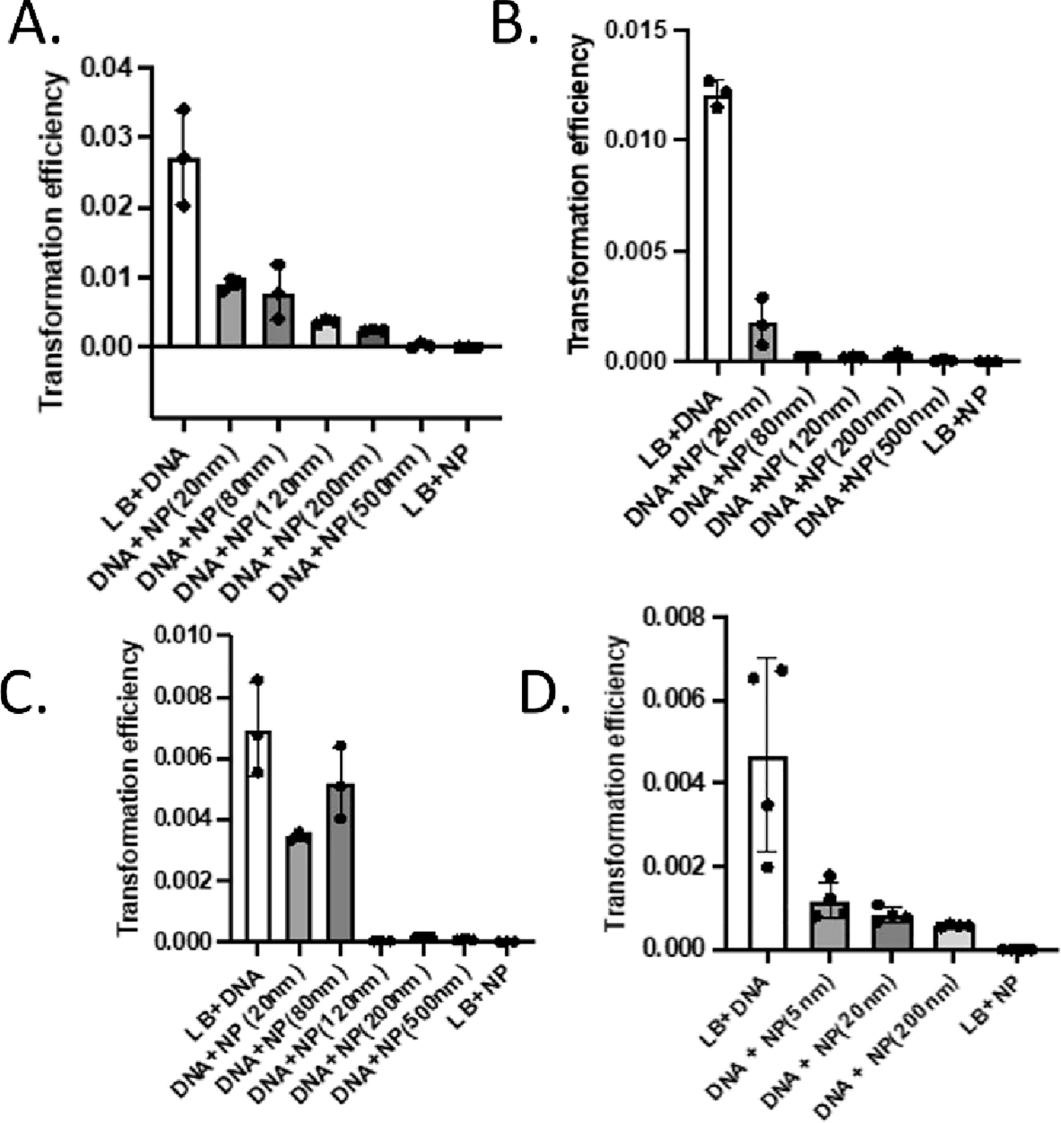
Reduction of *A. baylyi ADP1* transformation efficiency by silica and gold nanoparticles. In these experiments, all cells were cultured in LB Medium (LB). Positive controls are in the column (LB + DNA) and negative controls the last (LB + NP). (A) Size dependence of silica nanoparticle inhibition of transformation; smaller particles (columns 2 and 3: 20 and 80 nm SiO_2_NPs) show weak transformation compared to larger particles. (B) Increasing the NP-to-pBTK501 DNA ratio (DNA 1:3 SiO_2_NP) increases blocking of transformation with even smaller nanoparticles showing substantial blocking. (C) Increasing the pBTK501 DNA-to-NP ratio (DNA 3:1 SiO_2_NP) reduces the blocking of transformation by 20 and 80 nm particles, while all larger silica nanoparticles remain effective; (D) in *A. baylyi* ADP1 cultured in LB, all sizes of AuNPs tested reduce the transformation efficiency.

This inhibition of natural transformation by SiO_2_NPs is dose-dependent. When we decreased the ratio of DNA to NPs 3-fold, we observed more inhibition of transformation in both GL and LB medium with the smaller SiO_2_NPs (20–120 nm) (Figures 2B and 3B and Figures S2 and S3) than with an equal ratio of DNA to SiO_2_NPs. Increasing the ratio of DNA to NPs 3-fold resulted in the opposite result with less blocking of transformation and a general increase in transformation efficiency with SiO_2_NPs smaller than 120 nm (Figures 2C and 3C). In the GL experiments, the 20 nm SiO_2_NP treatment was not consistent with this trend, demonstrating that these conditions resulted in a significant reduction in transformation efficiency when compared to either the control, GL and DNA alone, or the 80 and 120 nm SiO_2_NPs (*p* < 0.05) (Figure 2C, second column). These results demonstrate that there is a dosage-dependent effect of SiO_2_NP inhibition of transformation. To determine whether this blocking of transformation was solely due to SiO_2_NPs or is also a property of other particles, we examined whether gold nanoparticles also blocked transformation. In these experiments, we exposed *A. baylyi* ADP1 cells cultured in either GL or LB medium to gold nanoparticles (AuNPs) of three diameters: 5, 20, and 200 nm. In these conditions, all three different-sized AuNPs demonstrated a significant reduction of transformation (Figures 2D and 3D) when compared to positive controls. In GL medium, we observed the most significant reduction with the 20 and 200 nm AuNPs, while in LB medium, we observed a similar trend (Figure 3D). We also examined whether transformation of linear DNA by *A. baylyi* ADP1 was blocked by SiO_2_NPs. To do this, we linearized the pBTK501 plasmid with a restriction enzyme, gel-purified the product, and tested it in our system. With all sizes of SiO_2_NPs in both culture media, we observed significant reduction of transformation events (Figure 4).

**Figure 4:**
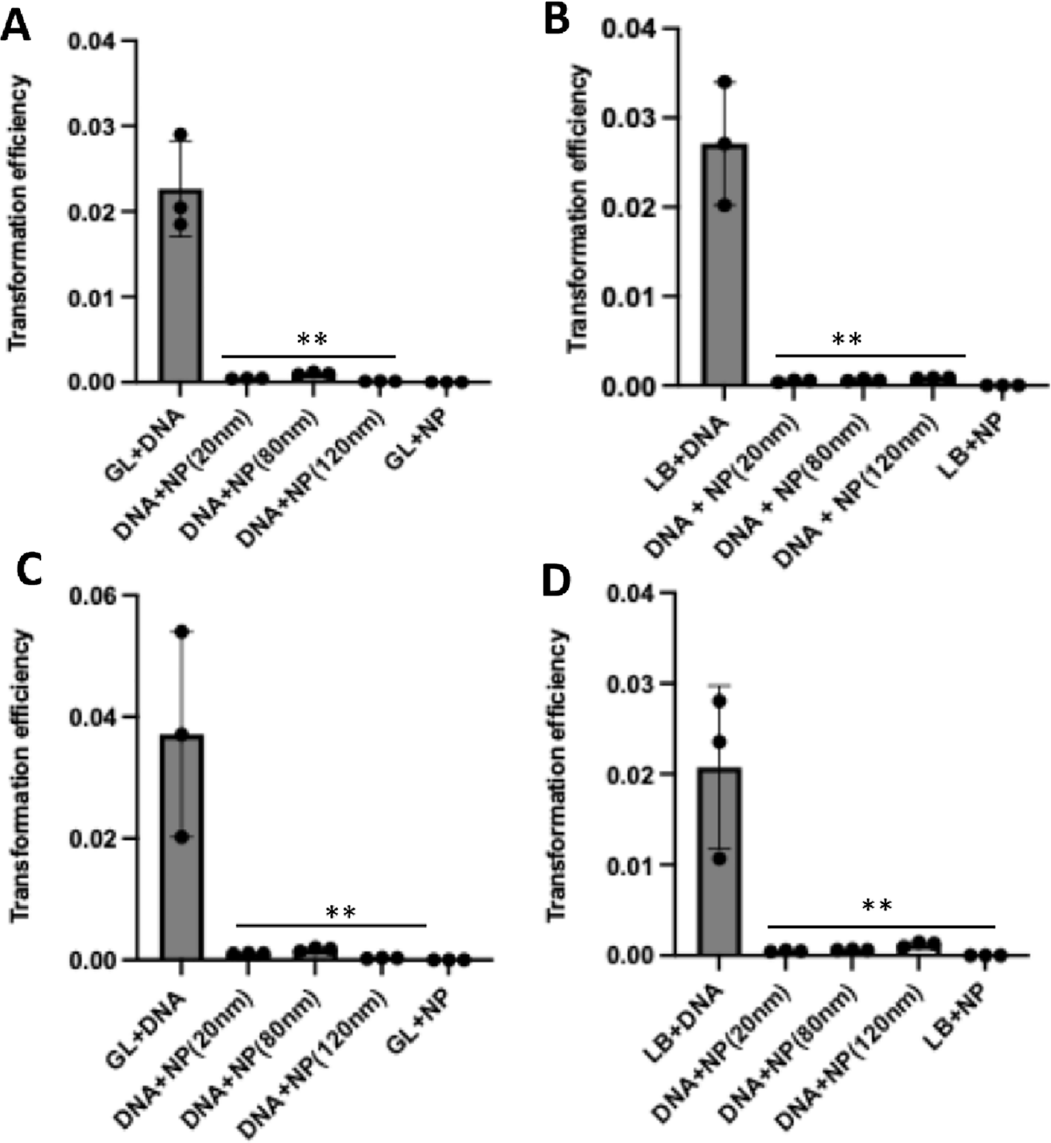
Size-independent inhibition of transformation by linearized pBTK501 DNA in *A. baylyi* ADP1 cultured in GL medium (A) and (B) LB medium (SiO_2_NP 1:1 DNA). (C) Transformation by linearized pBTK501 DNA in *A. baylyi* ADP1 cultured in GL medium (C) and (D) LB medium (SiO_2_NP 3:1 DNA). *p < 0.05 and **p < 0.01.

### Impact of SiO_2_NPs on Membrane Permeability, ROS Production, and T4P Pili Motility

To determine whether SiO_2_NPs altered the morphology of *A. baylyi* ADP1, we examined the cells using scanning electron microscopy and found that the morphology of *A. baylyi* ADP1 was unaffected by SiO_2_NPs (Figures S4 and S5). These results suggest that the cells were not damaged by SiO_2_NPs, thus reducing the transformation efficiency. In support of this observation, we saw no changes in membrane permeability of *A. baylyi* ADP1 cells exposed to SiO_2_NPs (Figure S6) Moreover, we observed no changes in *A. baylyi* ADP1 gliding motility in the presence of SiO_2_NPs, which is also linked to the T4P functionality (Figure S7). The production of ROS has been demonstrated to increase HGT and natural transformation.^29,25^ Increased production of ROS by natural organic materials exposed to simulated sunlight significantly enhances the natural transformation of *A. baylyi* ADP1.^30^ To determine whether reduced ROS production is responsible for this reduction in transformation, we examined whether the cells exposed to SiO_2_NPs exhibited changes to their redox state using H_2_DCFDA fluorescence. H_2_DCFDA is a fluorescent redox probe that when oxidized by free radical oxygen species, i.e., hydrogen peroxide (H_2_O_2_), increases its fluorescence.^31^ In cells treated with SiO_2_NPs, we observed a significant reduction of H_2_DCFDA fluorescence in cells exposed to SiO_2_NPs when compared to the positive controls (i.e., the addition of H_2_O_2_). SiO_2_NPs with diameters greater than 120 nm had a trend where the larger the particle, the lower the H_2_DCFDA fluorescence. In both growth conditions, the smallest 20 nm SiO_2_NPs showed no differences from the positive control, cells treated with 20 mM H_2_O_2_ (Figure 5A,B, second columns). In *A. baylyi* ADP1 cultured in LB with 80 nm SiO_2_NPs, we observed H_2_DCFDA fluorescence greater than that of this control (Figure 5B, third column). SiO_2_NPs induced an increase in ROS, which does not explain the reduction of transformation, and there was no correlation between levels of ROS production and changes to the transformation efficiency based on the size of the SiO_2_NPs.

**Figure 5:**
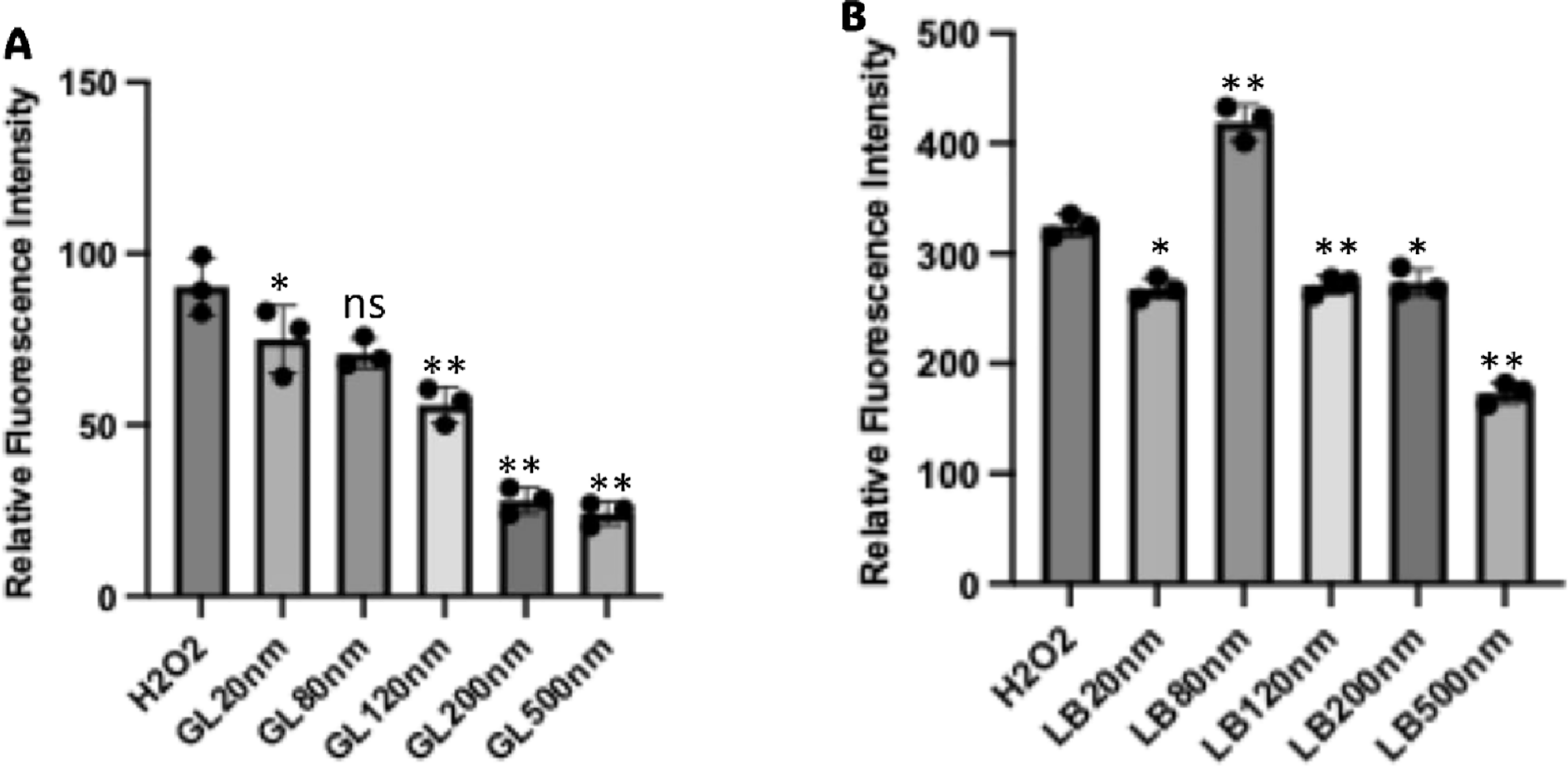
ROS production by exposure to SiO_2_NPs does not correlate with SiO_2_NP blocking of the transformation. Reactive oxygen species (ROS) results of *A. baylyi* ADP1 cells in the presence of silica nanoparticles. In each experiment, we used a positive control of 20 mM hydrogen peroxide in cells without NPs. (A) Relative H_2_DCFDA fluorescence intensity of *A. baylyi* ADP1 exposed to SiO_2_NPs cultivated in GL medium. There is a general downward trend of ROS production based on the NP size. (B) Relative fluorescence intensity of *A. baylyi* ADP1 cultivated in LB medium. Each treatment was analyzed in biological triplicate. Significant differences between nanoparticle-treated groups and the control (*A. baylyi* ADP1 wild type without nanoparticles) were tested with one-way ANOVA; *p < 0.05, **p < 0.01.

### SiO_2_NPs Blocks a T4P-Based eDNA Sensing Mechanism

Another means of reducing transformation would be to reduce the number of T4P since these cellular appendages are responsible for transduction of environmental DNA.^32,33^ To determine whether SiO_2_NP treatment influences the number of T4P pili, we used a genetically modified strain of *A. baylyi* ADP1 *TND0428* that has the S67C mutation in the pilA gene, which encodes the major structural component of T4P; the *pilA* S67C mutation enables labeling of the T4P via maleimide-based click chemistry^32^ and allowed us to use the Alexa488 fluorescence to quantify the amount of T4P per cell using flow cytometry. We tested the functionality of *A. baylyi* ADP1 *TND0428* T4Ps and found that these cells had slightly (∼2-fold lower) transformational competence (Figure S8); nevertheless, these results demonstrate that *A. baylyi* ADP1 *TND0428* T4Ps still transduce environmental DNA. In both culture media, we found that the presence of DNA (pBTK501) resulted in a slight but significant reduction of T4P and demonstrate that the presence of eDNA triggers a reduction in T4Ps possibly by eliciting a retraction of these structures (Figure 6A,B and Figure S9). When we added SiO_2_NPs to the *A. baylyi* ADP1 *TND0428* cells, we did not observe the characteristic reduction in the number of T4Ps, which suggests that these cells do not recognize or respond to the eDNA in cells cultured in either GL or LB media (Figure 6).

**Figure 6:**
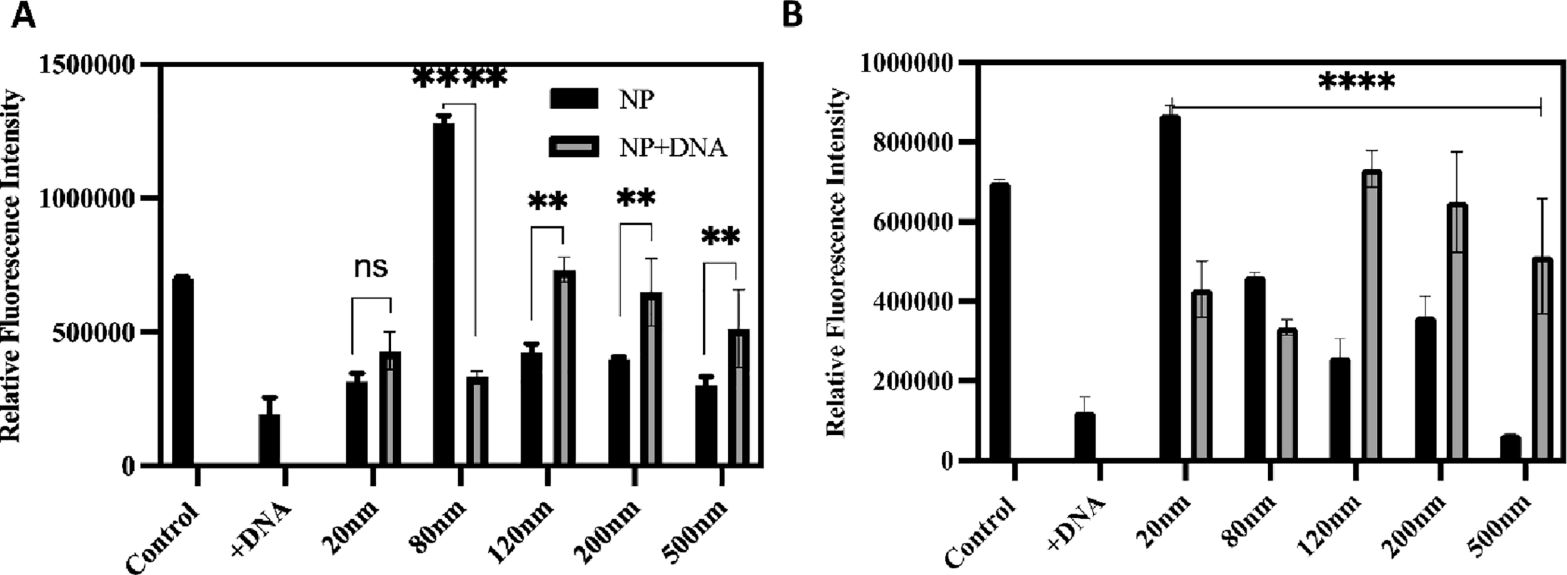
SiO_2_NPS prevents the DNA-dependent retraction of type IV pili (T4Ps). Graphs summarizing flow cytometry results of Alex488-labeled T4P in *A. baylyi* ADP1 *TND0428* cells with and without SiO_2_NPS and with and without eDNA­(pBTK501). In all experiments, the concentration of the nanoparticles was 20 μl/mL and the amount of DNA was 250 ng/mL. (A) *A. baylyi* ADP1 cultured in GL medium. (B) *A. baylyi* ADP1 cultured in LB medium. Fluorescence intensity is a function of the quality of the T4Ps. In no DNA controls (first column of either graph), we observed a level of fluorescence that is significantly reduced in the presence of the pBTK501 eDNA (second column of either graph). In the *A. baylyi* ADP1 incubated with all sizes of SiO_2_NPS, we observed either no significant reduction of T4P fluorescence in the presence of DNA or significantly more T4Ps in the presence of eDNA, which demonstrates that the DNA-dependent retraction/reduction of T4Ps does not occur in the presence of SiO_2_NPS. **p < 0.01, ***p < 0.001, and ****p < 0.0001.

### Environmental DNA and SiO_2_NP Interactions

These results suggested that the SiO_2_NPs sequester the eDNA, thereby preventing the cells from transformation (Figure 9). Silica binds DNA via a cooperative adsorption mechanism and has been used to purify DNA.^21^ Several studies have reported the shift in hydrodynamic volume in DNA and SiO_2_NP complexes to be due to the phosphate-silanol and hydrophobic interactions between the SiO_2_NPs and the DNA complex.^13,24,34^ We considered the possibility that the SiO_2_NPs sequester the DNA, thus preventing transformation. To determine whether the SiO_2_NPs bind the environmental DNA pBTK501, we used dynamic light scattering (DLS) to examine the change in the hydrodynamic size of the SiO_2_NPs used in this study before and after exposure to the DNA. We observed little changes in the polydispersity index, which demonstrates that the SiO_2_NPs remained dispersed despite the binding of DNA, as opposed to aggregating or precipitating SiO_2_NP/DNA complexes (Table 1). We also observed an increase in the hydrodynamic volume of the SiO_2_NP in the presence of pBTK501 DNA (Table 1) demonstrating that the presence of DNA changes SiO_2_NP sizes and suggesting that DNA is coating the particle surface. Supporting this idea, we observed that this increased volume reflects the possibility of containing more copies of the environmental pBTK501 DNA.

**Table 1:** Dynamic Light Scattering Measurement of Nanoparticles and DNA^a^

sample	***d*** **_h_** **(nm)**	PDI	zeta (mV)	volume (cubic nm)	change in volume with DNA	number of DNA copies in this volume
20 nm SiO_2_NPs	20.23 ± 0.2	0.19 ± 0.0	–41.75 ± 1.0	4.19 × 10^3^	NA	NA
80 nm SiO_2_NPs	102.4 ± 0.4	0.06 ± 0.0	–55.96 ± 1.0	5.56 × 10^5^	NA	NA
120 nm SiO_2_NPs	149 ± 0.4	0.07 ± 0.0	–49.26 ± 1.2	1.73 × 10^6^	NA	NA
200 nm SiO_2_NPs	228.3 ± 10.2	0.04 ± 0.0	–58.66 ± 0.6	6.21 × 10^6^	NA	NA
500 nm SiO_2_NPs	627 ± 30.4	0.05 ± 0.0	–73.35 ± 0.6	1.29 × 10^8^	NA	NA
DNA	37.56 ± 8.5	0.435 ± 0.2	–13.11 ± 0.1	2.77 × 10^4^	NA	NA
20 nm SiO_2_NPs + DNA	31.53 ± 10.4	0.52 ± 0.1	–9.93 ± 1.0	1.64 × 10^4^	1.22 × 10^4^	0.44
80 nm SiO_2_NPs + DNA	112.30 ± 0.8	0.06 ± 0.0	–29.81 ± 0.5	7.36 × 10^5^	1.80 × 10^5^	6.49
120 nm SiO_2_NPs + DNA	203.0 ± 4.3	0.15 ± 0.0	–33.66 ± 0.5	4.38 × 10^6^	2.65 × 10^6^	95.45
200 nm SiO_2_NPs + DNA	314.4 ± 17.7	0.17 ± 0.02	–42.92 ± 0.1	1.62 × 10^7^	1.00 × 10^7^	360.59
500 nm SiO_2_NPs + DNA	654.8 ± 9.65	0.05 ± 0.02	–36.03 ± 0.4	1.47 × 10^8^	1.79 × 10^7^	646.60

a Hydrodynamic volume (*d*_h_); polydispersity index (PDI); experiments were performed in biological triplicate.

We also observed a decrease in the zeta potential of SiO_2_NPs in the solutions. This result suggests that the DNA is shielding the surface of the SiO_2_NPs as the already negatively charged surface is now less negatively charged, closer to pure DNA (Table 1, last column). To confirm physical interaction between the SiO_2_NPs and the DNA, we used Fourier transform infrared (FTIR) spectroscopy to detect changes in the functional group bonds, specifically the phosphate groups of the DNA backbone. In our experiments, in both GL and LB media, we observed a significant increase in absorbance in the band reserved for the DNA phosphate group (1000–1100 cm^–1^)^35^ for 120 and 500 nm SiO_2_NPs when compared (Figure 7A,B), which is indicative of adsorption of pBTK501 to the SiO_2_NPs. UV/vis spectroscopy showed a similar trend that 500 nm SiO_2_NPs had the highest absorption at 300 nm, reinforcing the idea that the largest-size SiO_2_NPs have the highest level of DNA binding (Figure 8). These results along with the previous results suggest that SiO_2_NPs restrict access of *A. baylyi ADP1* to eDNA, thus resulting in a reduction in natural transformation.

**Figure 7:**
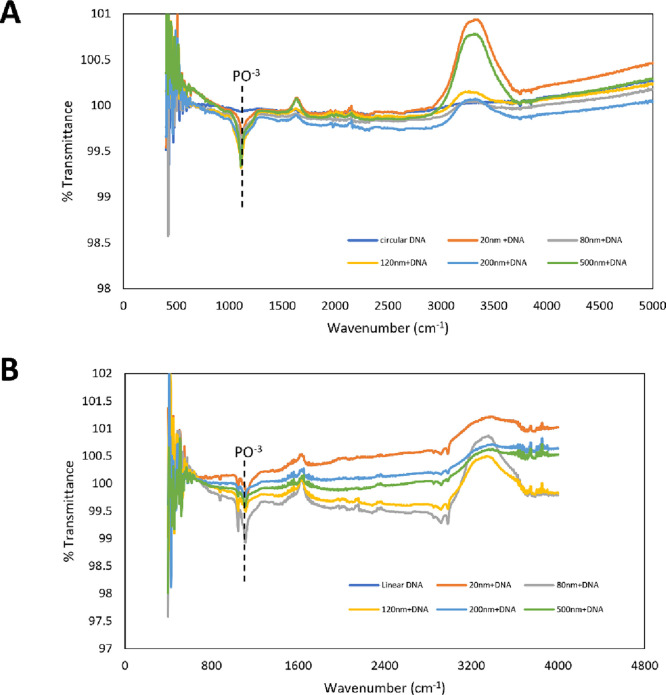
FTIR analysis of the interaction of the eDNA pBTK501 plasmid and the SiO_2_NPs used in this study. (A) Circular plasmid DNA PBTK501. (B) Linearized DNA of the same plasmid. The band around 1100 cm^–1^ reserved for phosphate groups (dashed line) in DNA showed a significant increase in absorbance with 120 and 599 nm SiO_2_NPs, which demonstrate a physical interaction between the DNA and SiO_2_NPs.

**Figure 8:**
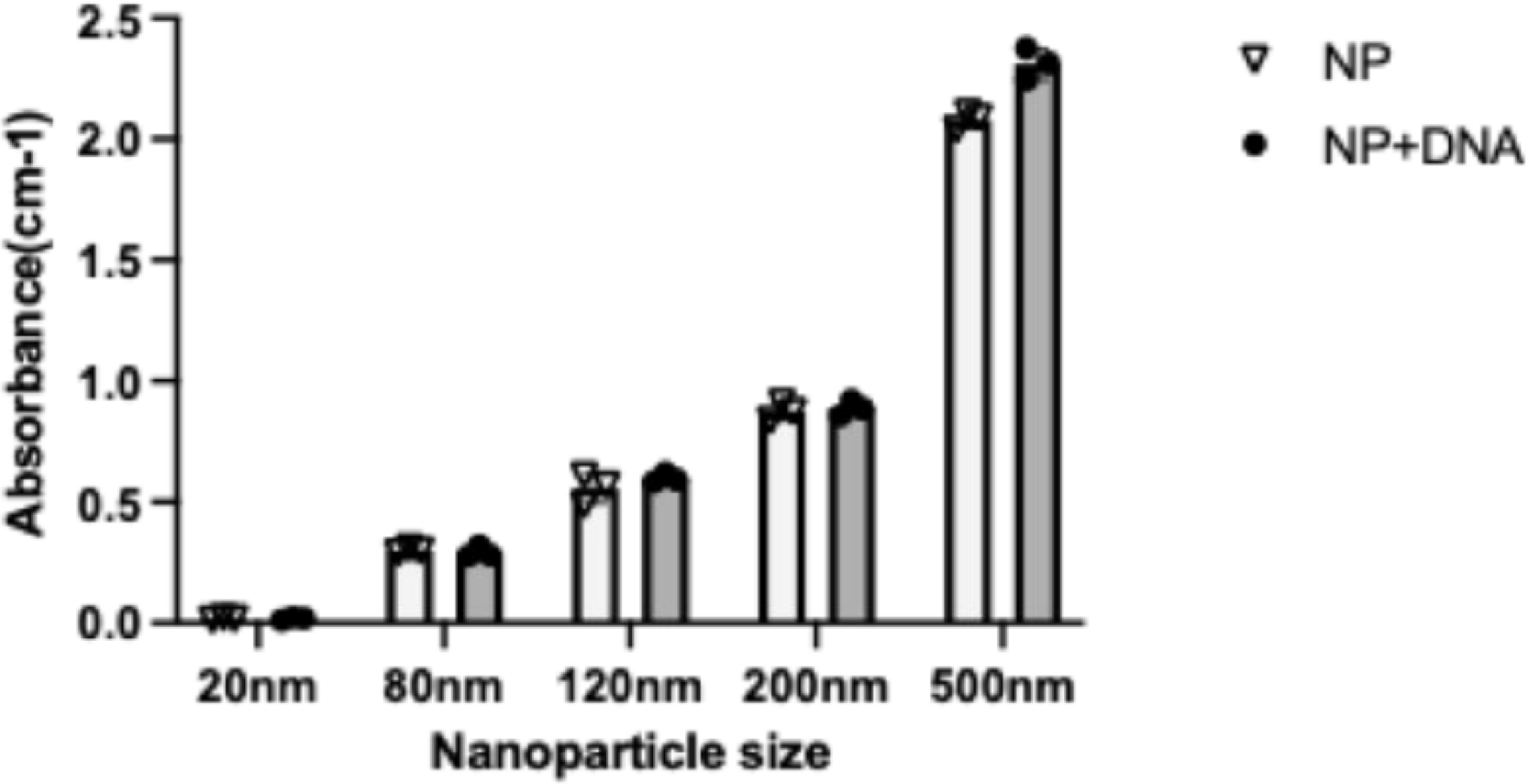
UV–vis spectra of the SiO_2_NP in the presence of pBTk501 DNA. Experiments were conducted in biological triplicate. *p* < 0.05.

## Discussion

Natural genetic transformation is the principal route by which antibiotic resistance persists and is spread throughout a microbial community.^38^ In the context of microbial populations, this sharing of genes increases the fitness of these microbes, especially in the context of drug resistance.^36,37^ Transformation is the principal route by which antibiotic resistance is shared and persists among microbial population.^38^ While much work has demonstrated HGT within the clinical system, recent work has demonstrated that HGT occurs in a range of environments including wastewater treatment plants, aqueous environments (e.g., rainwater runoff, groundwater, aquaculture, and surface water), and in the soil.^39–45^ External conditions such as the presence of heavy metals, organic molecules, and ROS-generating materials enhance HGT, usually by promoting DNA repair, innate stress responses, and/or alteration to metabolic processes.^30,46–51^ Farming is a major source for the generation and transmission of ARGs as farming uses large quantities of antibiotics and contains large and diverse microbial populations resulting in high levels of ARGs.^52^ Agriculture and farming, particularly the poultry industry, is dangerously poised to promote the rise and distribution of ARGS; these industries use 70% of the antibiotics produced, and the waste, manure, sewage, and soil surrounding poultry farms are considered hotspots for ARG generation and pollution.^39,53–56^

Current wastewater treatment while eliminating harmful bacteria may increase HGT by simultaneously killing microbial cells, thus releasing ARG DNA into the environment and enhancing HGT of the survivors.^54^ Furthermore, conditions in wastewater treatment and in other aqueous environments and challenges such as the presence of sludge and active biofilm formation make combating HGT difficult.^45,57^ The mitigation of environmental ARGs has focused on various physical and chemical treatments that either eliminated excess/waste antibiotics through processes such as pyrolysis, applications of probiotics, removing HGT triggers from the environment using adsorbents such as chitosan, or providing competing microbes that simultaneously promote natural degradation of ARGs and reduce the presence of pathogenic bacteria.^39,58,59^

Experimental methods using CRISPR-based technologies to engineer bacteria that are resistant to HGT are effective in a laboratory setting.^60^ HGT has a relationship with quorum sensing, the cell–cell communication that bacteria use to coordinate growth and metabolic processes; blocking quorum sensing blocks transformation. In *Streptococcus mutans*, mucin *O*-glycans suppress quorum sensing and transformation;^61^ these results suggest that transformation is tightly linked to metabolism and the microbial community as a whole. Similarly, in *Streptococcus pneumoniae*, small-molecule inhibitors called competence blocking compounds or COM-blockers disrupt the proton motive force interfering with quorum sensing and transformation.^62^ These results demonstrate the potential for external control over transformation using small-molecule drugs that target specific cellular mechanisms. While the methods described above reduce HGT and ARG in a variety of contexts, they may not be practical. Small organic compounds are susceptible to degradation and may be species-specific. Furthermore, the diversity of environmental conditions may also limit their utility.

Nanomaterials have been used to alter natural transformation. TiO_2_ nanoparticles (TiO_2_ NPs) inhibit natural transformation in the Gram-positive *Bacillus subtilis*,^25^ and although not natural transformation, TiO_2_ NPs significantly inhibited transformation in electro-competent *Escherichia coli* (*E. coli*) K12.^1^ In either case, the mechanism underlying the inhibition of transformation is not clear. Conversely, other nanomaterials have the opposite effect. Chitosan nanoparticles with a diameter of 125 nm enhanced transformation in *E. coli* K12,^26^ and ZnO NPs increase the transformation efficiency 3-fold in pure *E. coli* cultures.^27^ In another study, mesoporous silica nanoparticles measuring 2–50 nm in diameter enhanced sequence-specific transformations in *Neisseria meningitidis*.^28^ Transformation hinges on the ability of a bacterium to be competent and acquire external genetic elements such as plasmids and transposons, among other mobile genetic elements.

In this manuscript, we demonstrate that SiO_2_NPs reduce *A. baylyi* ADP1 transformation of both circular and linear environmental DNA regardless of the size of the SiO_2_NPs; however, larger SiO_2_NPs have a greater effect: 20 nm SiO_2_NPs reduced transformation efficiency by more than 2.3-fold, while the 500 nm SiO_2_NPs reduced the transformation event by 98-fold. Furthermore, the effect was dose-dependent; increasing the ratio of SiO_2_NPs to DNA resulted in a uniform increase in transformation. We speculate that the mechanism of SiO_2_NP action involves binding the DNA and thus preventing it from encountering the type IV pili (T4P) and that the interaction between the DNA and SiO_2_NPs is greater than the interaction between the DNA and T4P (Figure 9). However, we cannot dismiss the possibility that there are additional inhibitory interactions between the DNA/NP complexes and the T4Ps. All bacteria that engage in natural transformation use T4P for the binding and uptake of environmental DNA. T4Ps and their orthologs have been identified in many bacteria including many pathogenic bacteria such as *Streptococcus sanguinis*,^68^ *Clostridium* spp.,^66^ *Neisseria* spp*.*,^69^ *Vibrio* spp*.*,^70^ *Bacillus subtilis*,^66^ and *Enterobacteriaceae* such as *Citrobacter rodentium*.^71^ SiO_2_NPs may also block natural transformation in these pathogenic species as well, if the interactions between the SiO_2_NPs and DNA are greater than the DNA/T4P interaction. The identification of antitransformation materials that function by sequestering eDNA thus preventing natural transformation in a potentially general/nonspecies manner will have greater application and potential in controlling HGT and the spread of antibiotic resistance.

**Figure 9:**
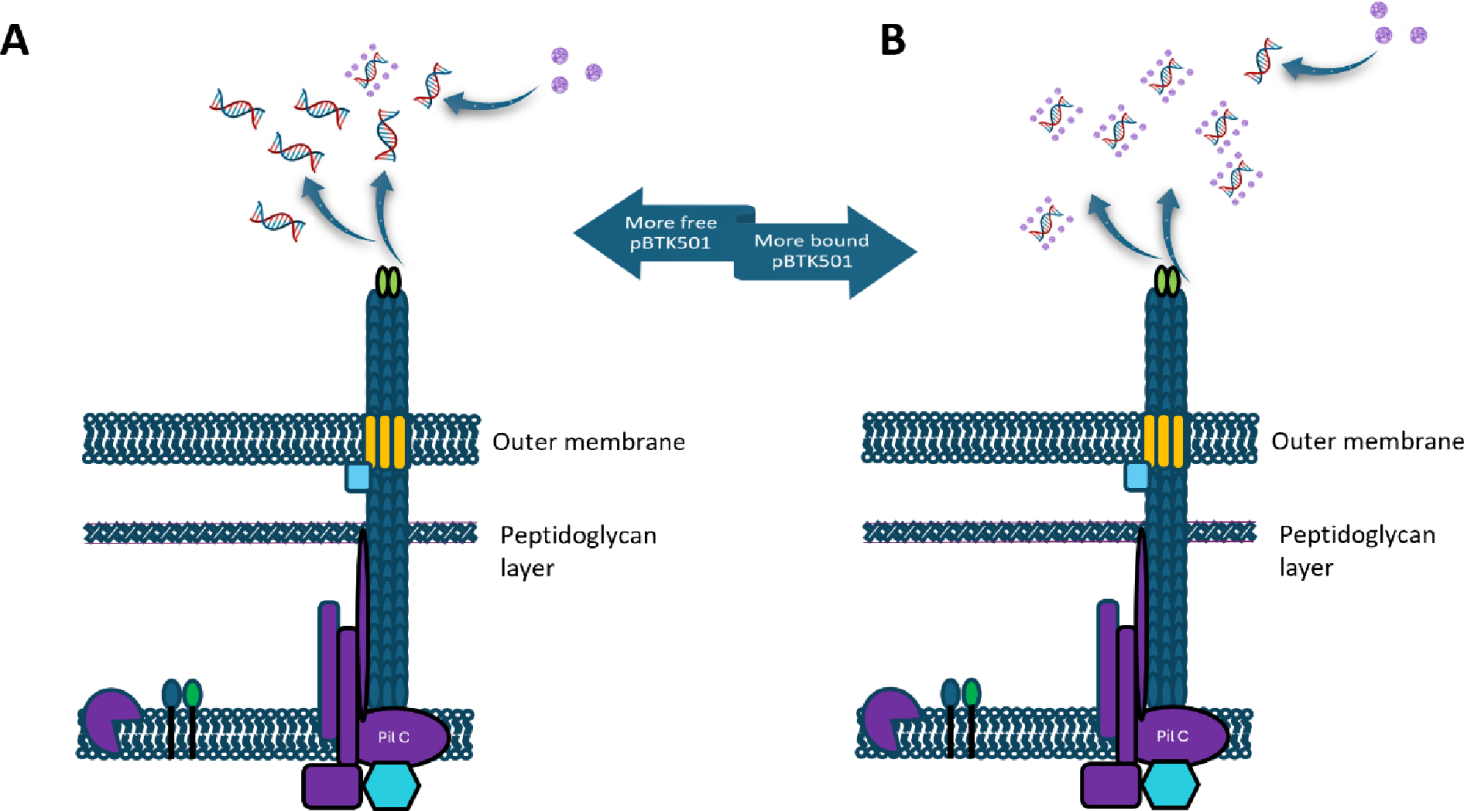
Proposed mechanism of action of SiO_2_NPs against *A. baylyi* ADP1 transformation efficiency. (A) pBTK501 binds to type IV pili of T4P of *A. baylyi* ADP1 for transformation to take place. (B) SiO_2_NPs bind to pBTK501 DNA, thus preventing the T4P of *A. baylyi* ADP1 from interacting with free pBTK501 DNA, leading to inhibition of transformation events. This reaction shows less free pBTK501 compared to bound pBTK501.

## Conclusions

Horizontal gene transfer (HGT), especially through natural transformation, is a key mechanism by which antibiotic resistance genes (ARGs) spread among microbial communities, often enhanced by environmental stressors such as heavy metals and reactive oxygen species. Agricultural practices, particularly in poultry farming, contribute significantly to ARG proliferation due to extensive antibiotic use and microbial diversity in waste and soil. Many antibacterial treatments such as those found in wastewater plants, while removing harmful bacteria, may inadvertently promote HGT by releasing ARGs into the environment, complicating mitigation efforts. Experimental approaches such as CRISPR and quorum sensing inhibitors show promise in reducing HGT, but their practical application is limited by environmental variability and compound degradation. We have shown that SiO_2_ nanoparticles inhibit transformation by binding DNA and preventing its uptake via type IV pili, suggesting a novel strategy for controlling ARG spread across diverse bacterial species.

## Materials and Methods

### Bacterial Culture

We used *A. baylyi* ADP1 (ATCC 33305) and *A. baylyi* ADP1 *TND0428,* which has the pilA S67C mutation to enable labeling the pili using click chemistry.^32^ We cultured our cells in two different media, LB^79,80^ and synthetic M9 media containing 2% glucose.^81^ For the transformation experiments, M9 minimal media was prepared with 2% glucose as the sole carbon source. All cultures were grown at 30 °C with 140 rpm shaking. For the transformation experiments, we used the plasmid pBTK501 (Addgene 110602) as our target; in these experiments, we used a 100 μg/mL concentration of ampicillin (Sigma 69-53-4). For transformation experiments using linear DNA, the plasmid pBTK501 was digested with *Eco*RI (New England Biolabs, R0101S) and the gel was purified prior to following the protocol as described in the kit (Invitrogen Purelink, K210012).

### Transformation Efficiency

Transformation efficiency was determined using the protocol described.^82^ For quantitative determination of transformation events, we plated 1 × 10^5^ cells of the mix on selective agar supplemented with 100 μg/mL ampicillin and the same on a nonselective agar plate for the enumeration of total CFU/mL. Transformation efficiency is defined as transformed CFU/mL divided by the total CFU/mL. We performed a fluctuation analysis to observe for spontaneous mutation. No spontaneous mutation was observed in multiple trials. For the positive control experiments, we added only plasmid pBTK501, while for the negative control, SiO_2_NPs were added without DNA.

### Nanomaterials

SiO_2_NPs were purchased from Nanocomposix of the following sizes: 20 (SISN20-25M), 80 (SISN80-25M), 120 (SISN120-25M), 200 (SISN200-25M), and 500 nm (SISN500-25M). We used three ratios of the number of SiO_2_NPs to the number of DNA molecules: 1:1, 3:1, and 1:3. To calculate these ratios, we used the following information. The number of DNA molecules was determined by considering the amount of DNA used in each experiment (250 ng) and the number of molecules per nanogram, as determined by the molecular mass of the pBTK501 plasmid. This number was then used to calculate the amount of SiO_2_NPs used, the number of SiO_2_NPs per unit volume (Table 2).

**Table 2:** Size and Surface Area of SiO_2_NPs Used in This Study

SiO_2_ nanoparticle size (nm)	concentration (mg/mL)	particle concentration (particles/mL)	surface area of SiO_2_NPs (nm^2^)	total surface area of 1 mL of SiO_2_NPs (nm ^2^)
20	10	1.1 × 10^15^	5024	5.57 + 18
80	10	1.7 × 10^13^	80,384	1.37 × 10^18^
120	10	5.0 × 10^12^	180,864	9.04 + 17
200	10	1.1 × 10^12^	502,400	5.57 + 17
500	10	7.1 × 10^10^	3,140,000	2.23 × 10^17^

### Stoichiometric Calculations

#### pBTK501 Plasmid DNA:Nanoparticles at 1:1

##### DNA Calculations

For example, we took 20 μL of the pBTK501 plasmid DNA to calculate the number of molecules present in the given volume.

The DNA concentration was obtained using a nanodrop spectrophotometer to be 245.3 ng/μL.

$$\mathrm{concentration}=\frac{\mathrm{mass}}{\mathrm{volume}}$$

Since we have a known concentration and volume, we substitute the (*C*) and (*V*) in the above equation to obtain the mass of the DNA in nanograms (ng).

Therefore, mass (*M*) = $C\times V=\frac{245.3\;\mathrm{ng}}{\mu L}\times 20\mu L=4906\;\mathrm{ng}$ .

We then convert the unit of mass to grams: 4.906 × 10^–6^ g.

The average mass of 1 bp dsDNA is 660g/mol.^83^

To obtain the total mass of the pBTK501 plasmid DNA from Addgene (plasmid #110602), with pBTK501 = 8801 bp, the total mass of the pBTK501 becomes $\mathrm{average}\;\mathrm{mass}\;\mathrm{of}\;1\;\mathrm{bp}\times 8801=\frac{660\;g}{\mathrm{mol}}\times 8801=5.81\times 10^{6}g/\mathrm{mol}$ .

$$\frac{4.906\;\times 10^{‐6}\;g}{5.81\;\times 10^{6}\;g/\mathrm{mol}}=8.45\;\times 10^{‐13}\;\mathrm{mol}$$

$$\mathrm{number}\;\mathrm{of}\;\mathrm{molecules}=8.45\;\times 10^{‐13}\;\mathrm{mol}\times \mathrm{Avogadro}’s\;\mathrm{number}$$

$$8.45\;\times 10^{‐13}\;\times 6.022\times 10^{23}=5.09\times 10^{11}$$

Therefore, the number of molecules in 20 μL of pBTK501 plasmid DNA is 5.09 × 10^11^ molecules.

##### Nanoparticle Equilibrium

Following Table 1 above, we determined that the number of molecules in 1 mL of solution in the 20 nm SiO_2_NPs is 1.1 × 10^15^.

We took 5 μL of the 20 nm SiO_2_NP solution, which contains 5.50 × 10^12^ molecules.

(i) Nanoparticle solution: 5.5 × 10^12^molecules in 5 μL.
(ii) pBTK501 plasmid DNA solution: 5.09 × 10^11^molecules in 20 μL.

To calculate the concentration of each solution, the concentration (*C*) is given by

$$C=\frac{\mathrm{number}\;\mathrm{of}\;\mathrm{molecules}}{\mathrm{volume}}$$

Therefore, the concentration of nanoparticles is

$$C_{20\mathrm{nm}}=\frac{5.5\times 10^{12}\;\mathrm{molecules}}{5\;\mu L}=1.1\times 10^{12}\;\mathrm{molecules}\;\mathrm{per}\;\mu L$$

For the concentration of pBTK501 plasmid DNA:

$$C_{\mathrm{pBTK}501}=\frac{5.09\times 10^{11}\;\mathrm{molecules}}{20\;\mu L}=2.545\times 10^{10}\;\mathrm{molecules}\;\mathrm{per}\;\mu L$$

We diluted the nanoparticle solution to be equal to the DNA molecules stoichiometrically:

$\mathrm{dilution}\;\mathrm{factor}\;(\mathrm{DF})=\frac{C_{20\mathrm{nm}}}{C_{\mathrm{pBTK}501}}=\frac{1.1\times 10^{12}}{2.545\times 10^{10}}=43.2$

Therefore, we diluted the nanoparticle solution 43.2 times to match the concentration of the pBTK501 plasmid DNA.

##### Dilution Setup

We did a single-step dilution at 1:43.2.

We diluted at 1:43.2, i.e., 1 μL of the nanoparticle solution was added to 42.2 μL of triple-filtered DI water. We vortexed it for 30 s.

The new concentration becomes 2.545 × 10^10^ molecules per μL.

##### Final Step

We pipet 20 μL of nanoparticle solution in a sterile 1.5 mL Eppendorf tube and added 20 μL of the pBTK501 plasmid DNA and gently swirled it up and down 10 times.

This mixture now contains an equal ratio of both the pBTK501 plasmid DNA and the nanoparticles.

#### DNA 1:3 SiO_2_

##### pBTK501 Plasmid DNA:Nanoparticles at 1:3

Both concentrations of nanoparticles from the initial stoichiometric calculations:

$$C_{20\mathrm{nm}}=\frac{5.5\times 10^{12}\;\mathrm{molecules}}{5\;\mu L}=1.1\times 10^{12}\;\mathrm{molecules}\;\mathrm{per}\;\mu L$$

For the concentration of pBTK501 plasmid DNA:

$$C_{\mathrm{pBTK}501}=\frac{5.09\times 10^{11}\;\mathrm{molecules}}{20\;\mu L}=2.545\times 10^{10}\;\mathrm{molecules}\;\mathrm{per}\;\mu L$$

##### Adjusting the Dilution Factor to 1:3

$$C_{20\mathrm{nm}}^{\prime }=3\times (2.545\times 10^{10})$$

$$C_{20\mathrm{nm}}^{\prime }=7.635\times 10^{10}\;\mathrm{molecules}\;\mathrm{per}\;\mu L$$

##### Dilution Setup

First, we calculated the dilution factor (DF):

$$\mathrm{DF}=\frac{C_{20\mathrm{nm}}}{C_{20\mathrm{nm}}^{\prime }}=\frac{1.1\times 10^{12}}{7.635\times 10^{10}}\approx 14.4$$

We did a single-step dilution at 1:14.4.

We diluted at 1:14.4, i.e., 1 μL of the nanoparticle solution was added to 13.4 μL of triple-filtered DI water. We vortexed it for 30 s.

The new concentration of the nanoparticle solution becomes 7.635 × 10^10^ molecules per μL.

##### Final Step

Now that the nanoparticle solution has 3 times the concentration of pBTK501 plasmid DNA, to ensure that the final number of molecules in each sample follows the 1:3 ratio, we used the same volume for both solutions (e.g., 20 μL each).

We pipetted 20 μL of nanoparticle solution into a sterile 1.5 mL Eppendorf tube and added 20 μL of the pBTK501 plasmid DNA and gently it swirled up and down 10 times.

This mixture now contains the pBTK501 plasmid DNA and the nanoparticles in 1:3.

#### DNA 3:1 SiO_2_

##### pBTK501 Plasmid DNA:Nanoparticles at 3:1

Both concentrations of nanoparticles from the initial stoichiometric calculations:

$$C_{20\mathrm{nm}}=\frac{5.5\times 10^{12}\;\mathrm{molecules}}{5\;\mu L}=1.1\times 10^{12}\;\mathrm{molecules}\;\mathrm{per}\;\mu L$$

For the concentration of pBTK501 plasmid DNA: $C_{\mathrm{pBTK}501}=\frac{5.09\times 10^{11}\;\mathrm{molecules}}{20\;\mu L}=2.545\times 10^{10}\;\mathrm{molecules}\;\mathrm{per}\;\mu L$

##### Adjusting the Dilution Factor to 3:1

$$C_{20\mathrm{nm}}^{\prime }=\frac{C_{\mathrm{pBTK}501}}{3}=\frac{2.545\times 10^{10}}{3}$$

$$C_{20\mathrm{nm}}^{\prime }=8.483\times 10^{9}\;\mathrm{molecules}\;\mathrm{per}\;\mu L$$

##### Dilution Setup

First, we calculated the dilution factor (DF):

$$\mathrm{DF}=\frac{C_{20\mathrm{nm}}}{C_{20\mathrm{nm}}^{\prime }}=\frac{1.1\times 10^{12}}{8.483\times 10^{9}}\approx 129.7$$

We did a double-step dilution since the nanoparticle solution needed to be diluted approximately 130 times.

(i) We pipetted 1 μL of nanoparticle solution into a sterile 0.6 mL Eppendorf tube, added 9 μL of the triple-filtered DI water, and gently swirled it side to side 10 times. The new concentration becomes 1.1 × 10^11^ molecules per μL.
(ii) We pipetted 1 μL of nanoparticle solution from step (i) into a sterile 0.6 mL Eppendorf tube, added 12 μL of the triple-filtered DI water, and gently swirled it side to side 10 times. The new concentration becomes 8.483 × 10^9^ molecules per μL.

##### Final Step

Now that the pBTK501 plasmid DNA solution has 3 times the concentration of nanoparticles, to ensure that the final number of molecules in each sample follows the 3:1 ratio, we used the same volume for both solutions (e.g., 20 μL each).

We pipetted 20 μL of nanoparticle solution into a sterile 1.5 mL Eppendorf tube and added 20 μL of the pBTK501 plasmid DNA and gently swirled it side to side 10 times.

This mixture now contains the pBTK501 plasmid DNA and the nanoparticles in 3:1.

So, the nanoparticle solution becomes 8.483 × 10^9^ molecules per μL × 20 μL = 1.697 × 10^11^ molecules.

Again, the pBTK501 plasmid DNA solution becomes 2.545 × 10^10^ molecules per μL × 20 μL = 5.09 × 10^11^ molecules.

### Dynamic Light Scattering (DLS) and UV–Vis Spectroscopy

Dynamic light scattering experiments were carried out using a Malvern Zetasizer Ultra (Malvern Panalytical Ltd., Spectris Company, Worcestershire, United Kingdom) with a 10 (MW) helium–neon (HeNe) laser having a light source of 633 nm. SiO_2_NP samples were dispersed in deionized water. The measurements were performed at a scattering angle of 173° and a temperature of 25 °C. The processing of the DLS raw data was performed using Excel software (Microsoft, Inc.). For calculation of particle sizes, standard values of viscosity and the refractive index of deionized water and SiO_2_NPs were used. For each sample, the data set from 5 measurements was averaged. The equilibration time for one measurement was 120 s. The hydrodynamic radius (*d*_h_) and zeta potential were obtained. Ultraviolet spectroscopy of the samples was carried out using a Nanodrop 2000 (Thermo Fisher). The UV–visible spectra were acquired between 200 and 750 nm.

### Click Chemistry and Flow Cytometry

To quantify changes in T4P levels, we used the following methodology described with some modification.^32^ In brief, 100 μL of *A. baylyi* ADP1 *TND0428* cells was cultured in various control and experimental conditions, i.e., either LB or GL, with and without SiO_2_NPs and with and without DNA to an OD of 0.3–0.6. Cells were then centrifuged and then resuspended in 100 μL of 1× PBS. Alexa Fluor 488 C5 maleimide (ex 495/em 519; Thermo Fisher A10254) was added to these cells to 25 μg/mL and incubated at room temperature for 30 min; then, labeled cells were centrifuged, washed five times with 1× PBS, and resuspended in 300 μL of PBS. The fluorescence intensity of the labeled *A. baylyi* ADP1 cells T4P was measured using a CytoFLEX flow cytometer (Beckman Coulter, USA). T4P density was assessed quantitatively by measuring fluorescence intensity. Flow cytometric acquisition parameters were standardized with a flow rate of 10 μL/min, and event detection was calibrated to approximately 150 events/s. Each sample analysis incorporated approximately 3000 cellular events. To ensure robust statistical inference, all experimental conditions were evaluated in biological triplicates.

### Fourier Transform Infrared Spectroscopy of SiO_2_NPs

The spectra in the mid-infrared section (4000–400 cm^–1^) of the electromagnetic spectrum were used to obtain the Fourier transform infrared spectroscopy (Thermo Scientific Nicolet iS50 FTIR spectrophotometer, Waltham, Massachusetts, USA) results of the shift in the DNA functional group band with and without SiO_2_NPs.

### Reactive Oxygen Species

The level of reactive oxygen species (ROS) was quantified following the method described by ref 84 with slight modification. The sensitivity of bacterial cells to Si0_2_NPs was assessed using the oxidant-sensitive fluorescent probe 2′,7′-dichlorodihydrofluorescein diacetate (H_2_DCFDA; Thermo Fisher, D399). The bacterial cultures were grown in either LB or GL media to an OD_600_ of 0.4. H_2_DCFDA at a concentration of 20 mM dissolved in dimethyl sulfoxide (DMSO) was added to the cellular suspension at a dilution of 1:2000, and the suspension was incubated at 37 °C with agitation for 30 min. Hydrogen peroxide (H_2_O_2_) was used as a positive control to trigger ROS production in the cells. The fluorescence intensities of the oxidized product 2′,7′-dichlorofluorescein (DCF) were measured using a fluorescence spectrophotometer (Synergy H1, Biotek) with the excitation and emission wavelengths of 488 and 535 nm, respectively.

### Statistical Analysis

All of the experiments were conducted independently, at least in biological triplicate. All data were expressed as means ± SD and were analyzed with Prism (GraphPad Software, Inc., California, USA). Significant differences were performed by the one-way ANOVA test; *p* values less than 0.05 were statistically significant.

## Supplementary Material



## Notes

Materials, growth curve of the *A. baylyi* ADP1 in both LB and M9 media (Figure S1), transformation on selective plates (Figures S2 and S3), SEM images (Figures S4 and S5), cell permeability data (Figure S6), pili quantification (Figure S7), wild type vs mutant transformation (Figure S8), and DNA sensing data (Figure S9) (PDF)
