# Supplementary material for: Silica Nanoparticles Block Natural Genetic Transformation in Acinetobacter baylyi ADP1
Source: ACS Omega. 2025 Dec 15;10(51):62609–20. doi: 10.1021/acsomega.5c06566 (PMC12756840; doi:10.1021/acsomega.5c06566)
Supplement: Supplementary file 1 [file ao5c06566_si_001.pdf]

Supplementary File
Title: Silica Nanoparticles Block Natural Genetic Transformation in *Acinetobacter baylyi* ADP1

Authors: Samuel Chetachukwu Adegoke, Ignatius Senyo Yao Yawlui, Dennis LaJeunesse*
Affiliation: Joint School of Nanoscience and Nanoengineering, Department of Nanoscience. E Gate city BLVD, Greensboro, North Carolina, USA

*Author for correspondence: drlajeun@uncg.edu, (336)285-2866

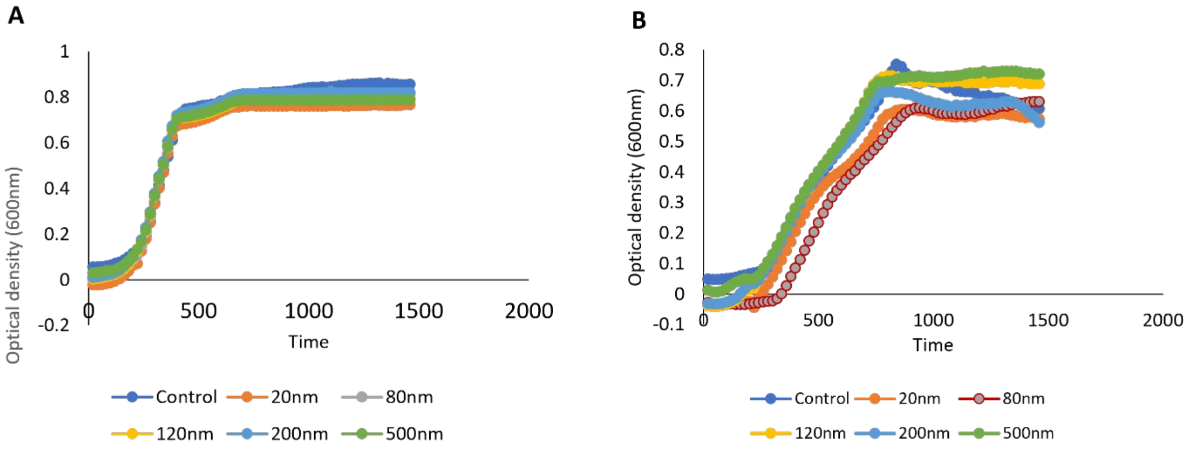

Figure S1. (A) Growth curve of *A. baylyi ADP1* in LB media with SiO_2 nanoparticles. (B) growth curve of *A baylyi ADP1* in GL media with SiO_2 nanoparticles.

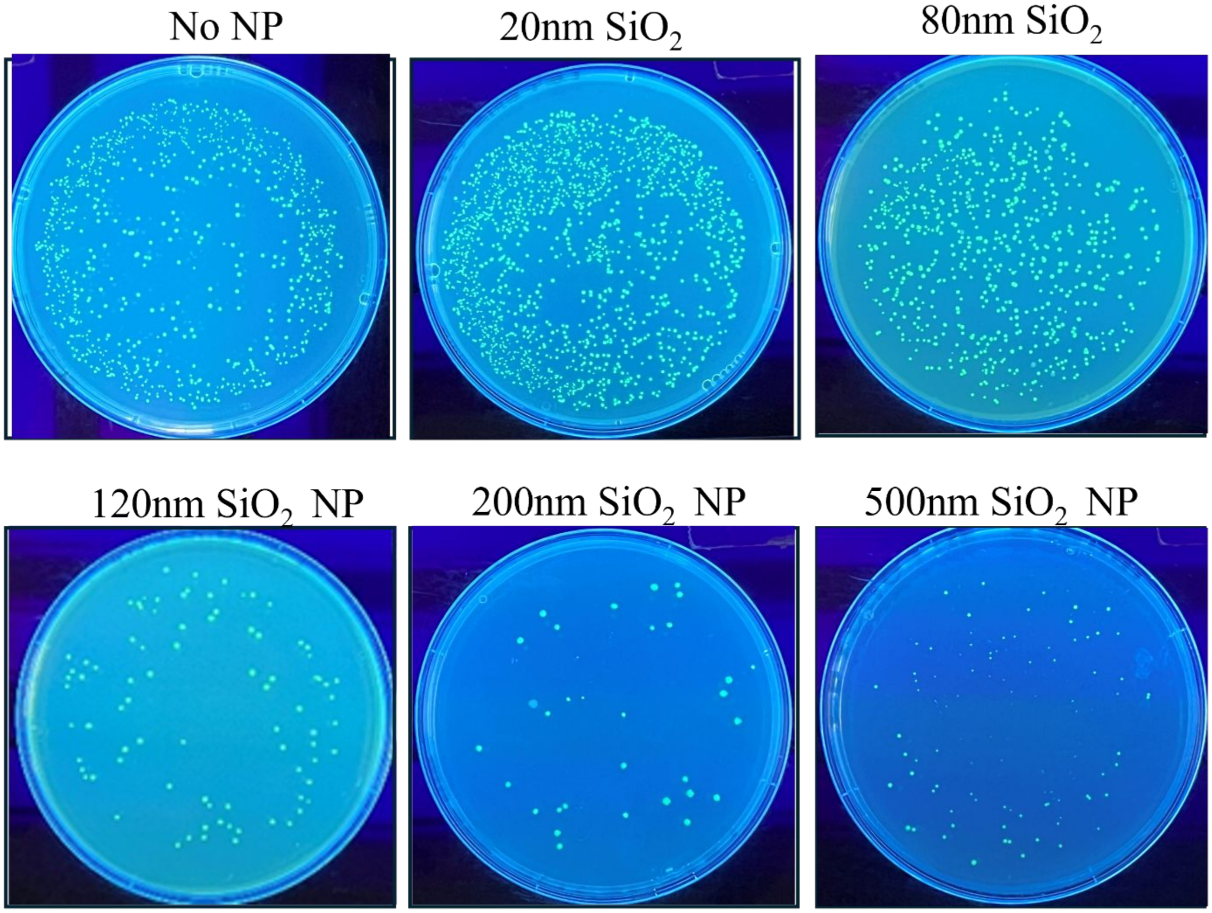

Figure S2. Examples of colonies counts on selective agar plates demonstrating the transformation efficiency of *A. baylyi ADP1* grown in LB medium at DNA to NP ratio of 3:1. The control sample without nanoparticles (upper row left) displays higher transformation rates compared to the SiO_2NP. Also, the 200 nm and the 500 nm showed reduced rate of transformation.

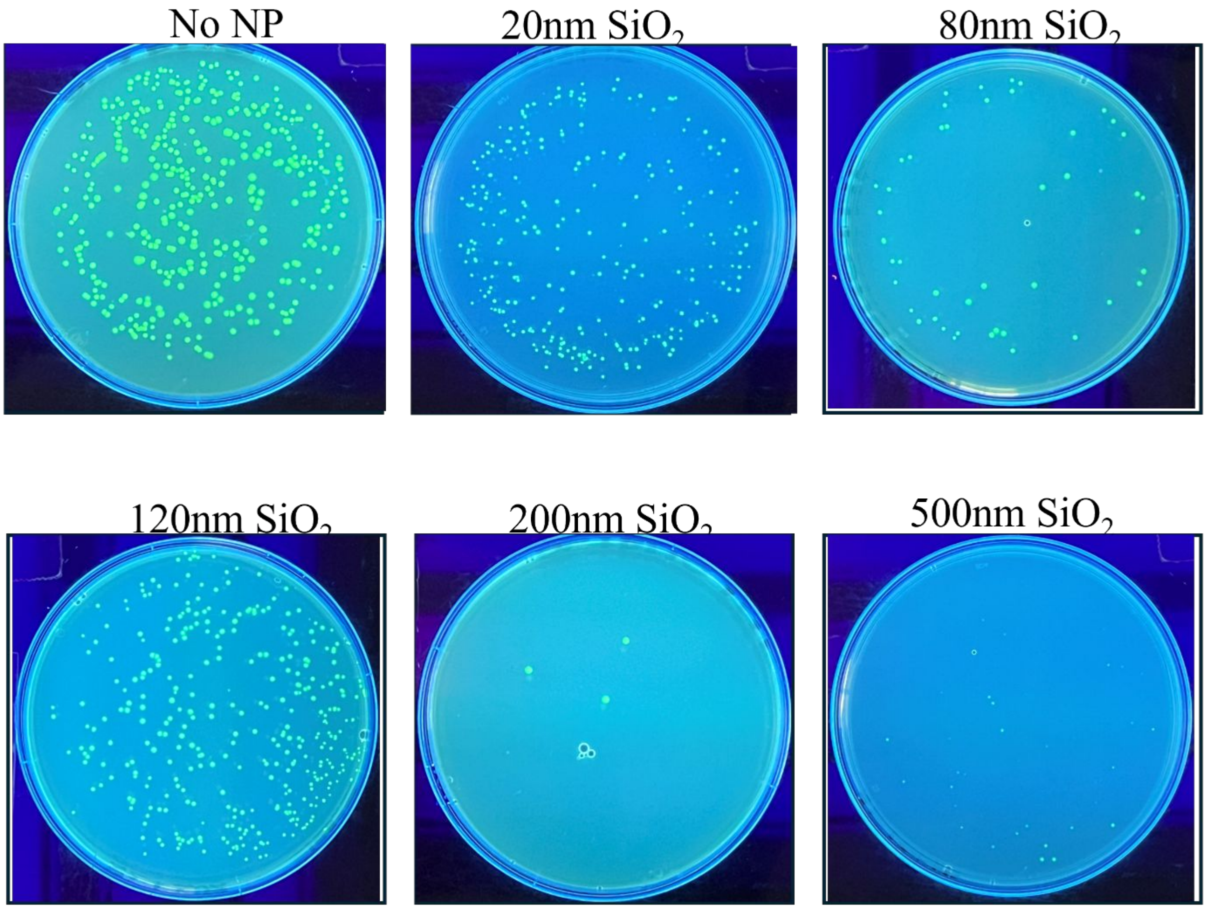

Figure S3. Examples of colonies counts on selective agar plates demonstrating the transformation efficiency of *A. baylyi ADP1* grown in controlled medium (M9 supplemented with 2% glucose) at DNA to NP ratio of 3:1. The control sample without nanoparticles (upper row left) displays higher transformation rates compared to the SiO_2NP. Also, the 200 nm and the 500 nm showed reduced rate of transformation.

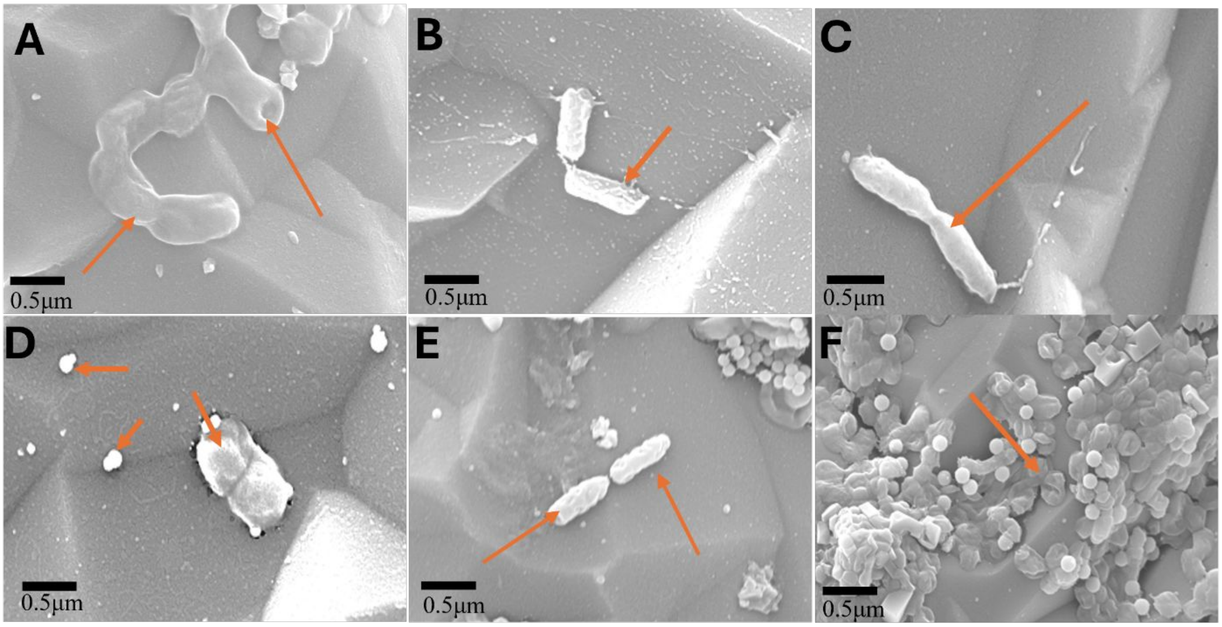

Figure S4. SEM images of *A. baylyi ADP1* in the presence of SiO_2NP of varying sizes. The cells were grown in M9 media supplemented with 2% glucose as the sole carbon source. One common feature conspicuously missing is the pili structure. All scale bars are 500 nm. In the nanoparticles perturbed samples, it appears that the nanoparticles were physically interacting with the cells (20 nm - 500nm). A) No NP added, cells are cylindrical; no pili is visible on the cells surface (Red arrow); B) 20 nm SiO_2NP treatment; C) 80 nm SiO_2NP treatment; D) 120 nm SiO_2NP treatment ; E) 200 nm SiO_2NP treatment ; and F) 500 nm SiO_2NP treatment.

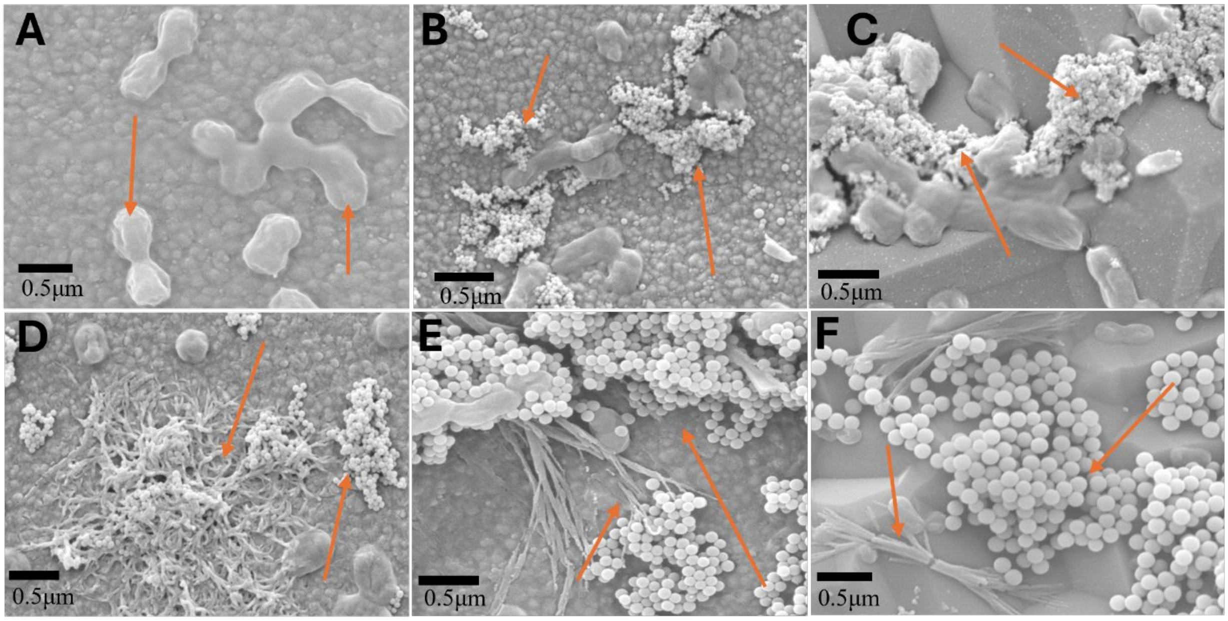

Figure S5. SEM images of *A. baylyi ADP1* in the presence of SiO_2NP of varying sizes. The cells were grown in LB media. One common feature is the pili structure. All scale bars are 500 nm. In the nanoparticles perturbed samples, it appears that the nanoparticles were physically interacting with the pili (20 nm - 500nm). A) No NP added, cell are contoured and no pili is visible on the cells surface (Red arrow); B) 20 nm SiO_2NP treatment; C) 80 nm SiO_2NP treatment; D) 120 nm SiO_2NP treatment ; E) 200 nm SiO_2NP treatment ; and F) 500 nm SiO_2NP treatment.

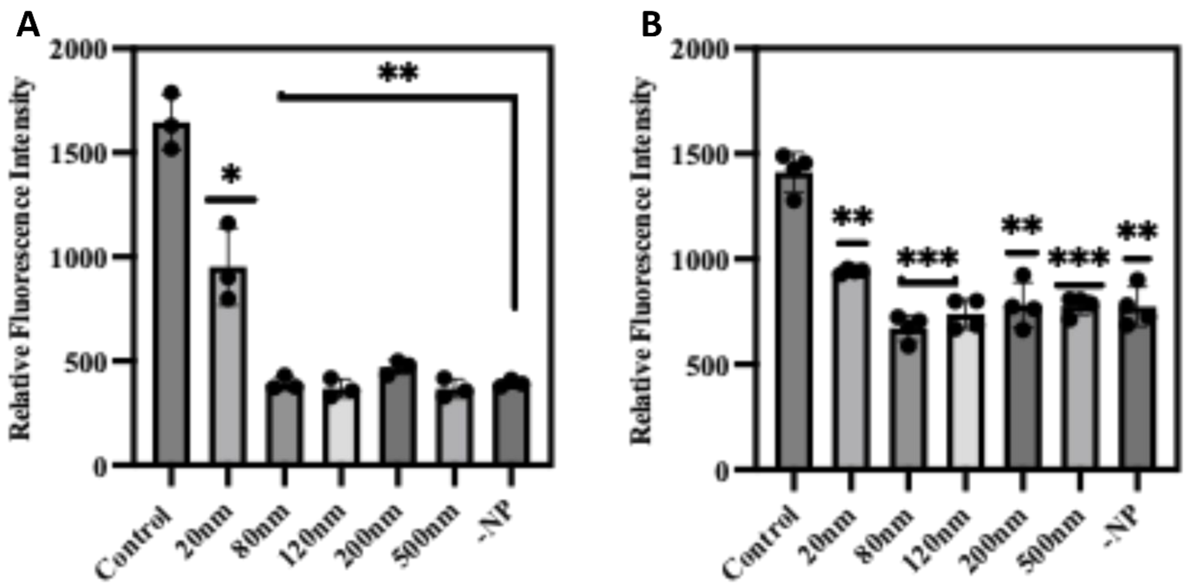

Figure S6. No change in cell permeability of *A baylyi ADP1* in the presence of SiO_2NPs. *A. baylyi ADP1* cells labeled with the fluorescent probe propidium iodide (PI). **A.** Cell permeability of *A. baylyi ADP1* cultivated in LB medium. **B.** Cell permeability of *A. baylyi ADP1* cultivated in GL medium. Positive control (cells heated to 60°C for 10 mins), negative control (-NP) cells without nanoparticle treatment. In both media, there is significant difference between NP treated cells and the control. Significant difference between nanoparticle treated groups and the control were tested with paired t-test, *p<0.05, ** p<0.01, ***p< 0.001.

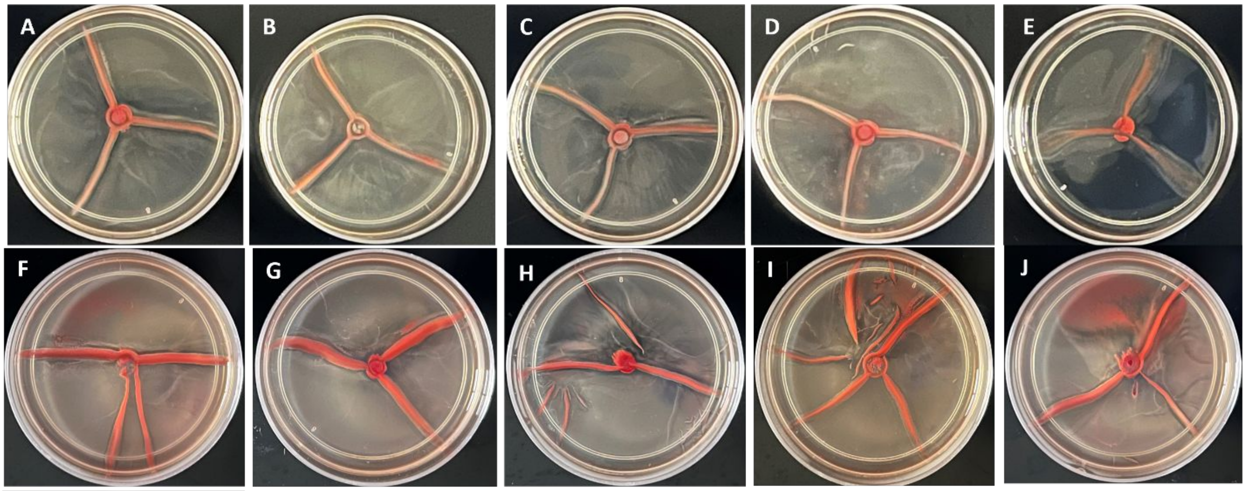

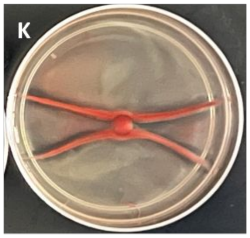

Figure S7. Effect of SiO_2 nanoparticle on the twitching of *A. baylyi ADP1*, (A). Control (no nanoparticle, *A. baylyi ADP1* cultivated in M9+2% glucose). (B). GL20nm, (C). GL80nm, (D). GL200nm, (E). GL500nm), (F). LB control (no nanoparticle, *A. baylyi ADP1* cultivated in LB), (G). LB20nm, (H). LB80nm, (I). LB120nm, (J). LB200nm, (K). LB500nm.

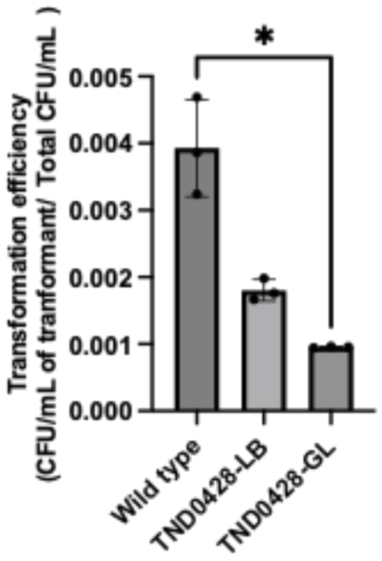

Figure S8*. A. baylyi ADP1* TND0428 is competent for transformation. *A. baylyi ADP1* (wild type) cells demonstrates a transformation efficiency of 4 transformants per cell, while *A. baylyi ADP1* TND0428 have a reduced transformation of 2/1000 and 1/1000 when cultured in LB or GL media respectively.

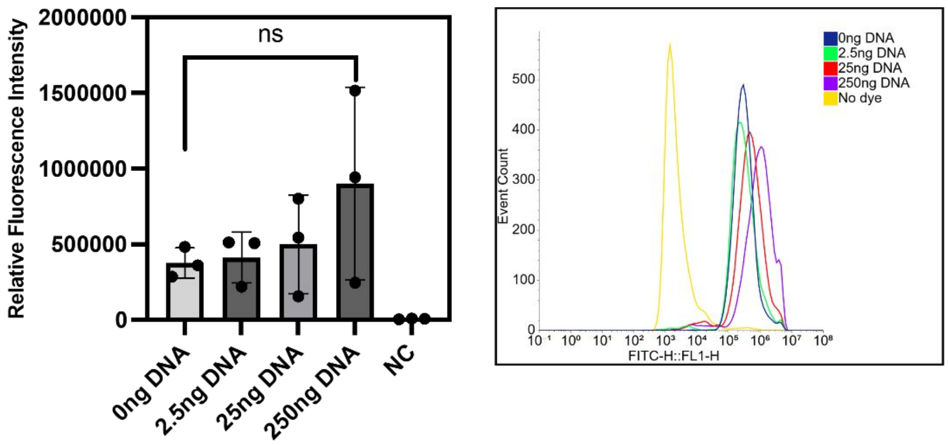

Figure S9. pBTK501 sensing study of Type IV pili (T4P) in *A baylyi ADP1*. *N*egative control (NC) cells without DNA & dye treatment, there is no significant difference between the 0ng, 2.5ng, and the 25ng DNA treatments. However, the 250ng treatment seems to trigger more cell T4P biosynthesis.
